# Martha’s Rule and the perioperative team: What changes, and what still needs to

**DOI:** 10.1177/17504589261449155

**Published:** 2026-06-17

**Authors:** Douglas Blackwood

In September 2022, Merope Mills published an account in *The Guardian* of the death of her 13-year-old daughter, Martha, at King’s College Hospital the previous summer (Mills 2022). Martha had been admitted after a handlebar injury to her pancreas. She developed sepsis and died, despite her mother’s repeated warnings that she was deteriorating. An inquest ruled that Martha would probably have survived had she been moved to intensive care earlier (Hassell 2022). Martha’s Rule is the policy response, expanded to include all English acute inpatient sites from September 2025 (NHS England 2025).

The policy has three components. First, any member of staff may request review from an independent team, typically critical care outreach, if they believe a deteriorating patient is not being responded to. Second, patients, families, carers and advocates may trigger the same 24/7 review through a mechanism advertised around the hospital. Third, every inpatient must be asked daily whether they are getting ‘better or worse’, with the response documented and acted upon (NHS England n.d., Welch et al 2025). All three components must be in place across the English acute sector by 31 March 2027, mandated through the NHS Standard Contract 2026/2027 (NHS England n.d.).

For those of us caring for patients after surgery, the situation Martha’s Rule attempts to address is well described. A patient comes through theatre and recovery, then moves to a general ward. In the days that follow, some deteriorate. A proportion need unplanned admission to intensive care and a smaller number die. The medical literature calls this pattern ‘failure to rescue’. The key point is not the complication itself. The difference between the number of complications in the best and the worst hospitals is relatively small. However, what varies to a much greater degree is what happens when a complication does occur (Ghaferi et al 2009). In the best hospitals, deterioration is identified quickly and treated. In others, it is missed or the response delayed, and the patient dies of something that might have been survivable. Martha’s Rule aims to prompt a faster response to deterioration.

The third component, the daily wellness question, asks each inpatient whether they are getting better or worse. The question and the requirement to document and act on the response are nationally mandated, although the specific response pathway is set locally by each trust. Recent NHS England (2026) data show that where the question has been the reason for an activation, 67% of calls led to a change in treatment, and a further 14% to a transfer of care. However, the wellness question may be less informative for surgical patients than for medical ones. After an operation, pain, nausea and fatigue are expected. The inflammatory response peaks around 48 to 72 hours after surgery, and feeling worse may be the norm rather than a warning sign. Unfortunately, NHS England’s published data does not yet differentiate between surgical and medical patients, or by specialty, which would help us understand the nuances of different patient cohorts.

It is worth remembering that Martha herself was being cared for on a surgical ward. Any member of staff, or family member, can, and should, request review from an independent team when their concerns have not been acted upon. However, in recovery, the post-anaesthesia care unit or an enhanced-care ward, the picture is slightly different. A consultant anaesthetist, surgeon or intensivist is usually close-by and families are often not present. In addition, physiology and subjective feeling are frequently abnormal due to the nature of surgery and anaesthesia. What the staff and family branches of Martha’s Rule means in those settings, and to whom one would be escalating, is not straightforward.

Martha’s Rule has been criticised for being introduced at scale without a strong evidence base, with its effectiveness evaluated retrospectively. That is true, but the policy is now mandated across every acute NHS trust in England. If the policy is to make the greatest difference, we need to record actively how it works in our specific setting, and to ask NHS England for data stratified by patient cohort.


Douglas Blackwood University College London, Faculty of Medical Sciences, London, UK Email: douglas.blackwood.16@ucl.ac.uk

